# A Report on Typhoid Fever Made to the Scott County Medical Society

**Published:** 1850-09

**Authors:** W. L. Sutton

**Affiliations:** Georgetown, Ky.


### WESTERN JOURNAL

OF

### MEDICINE AND SURGERY.

---

SEPTEMBER, 1850.

---

ART. I.—*A Report on Typhoid Fever made to the Scott County Medical Society*. By W. L. SUTTON, M. D., of Georgetown, Ky.

### INTRODUCTORY.

A report on typhoid fever made to the Scott County Medical Society, at their annual meeting in April, 1846, in accordance with the constitution of that Society, was the origin of this essay. Subsequently, I reflected that as this form of disease is at present attracting much attention on the part of our profession; that if not a new disease among us, some very important features in it have been overlooked; that in various portions of the West and South, it does not exist, or has not been detected; that it is very important, in this transition stage of our knowledge of this subject, that all the means which can be made available towards forming a fixed and correct opinion, should be within reach; and that a true delineation of this form of disease, as it appears at different times and places, drawn by faithful observers, is very important to coming to correct notions as to its nature, causes, and treatment; I have determined to discharge what I conceive my duty to the profession, and give an account of it, as it appeared in Georgetown and its vicinity, for the last six years. My remarks are not intended to apply to any other time or place. I have depended mainly upon my own observations as to facts. I have taken considerable pains to make myself acquainted with the circumstances attending these facts; so as to be better enabled to judge what influence these circumstances exerted upon conclusions to be drawn from these facts.

I have endeavored to observe correctly, and to report faithfully. I have no favorite theory to maintain; and if I had, I should desire a better basis than facts badly observed, or unfairly stated. I have conferred freely with my professional brethren, and have their general concurrence as to the correctness of my observations. Facts, not of my own observation, or which go to militate against those, I have given on the authority of the parties furnishing them. Of course I have introduced none which I do not consider fully entitled to credit.

In considering the etiology, and pathology, I have availed myself of the labors of other men, usually in their own words, that no change of meaning might be given to their facts or opinions, by change of language.

In my interpretations of those facts and opinions, I have endeavored to follow where reason and common sense led the way. I have not attempted to give a complete account of the history, causes, symptoms, or treatment of the disease, or form of disease as it may be. I do not pretend to have settled points in its etiology, pathology, or treatment; but I *have* aimed to give a correct account of its symptoms and treatment as observed here.

Upon some points it may be thought that I have expressed myself too strongly, and it may be so. I know that it is extremely difficult to be sure that we are in possession of all the facts in any given case. I know too, that I am as liable to overlook facts and circumstances as others. I can only say, if I have drawn incorrect conclusions, no man will regret it more than myself.

From the fact that I have quoted pretty freely from Prof. Bartlett's work on typhus and typhoid fevers, and in some instances have viewed his facts in a light different from that in which he views them, it may be supposed I take pleasure in differing from him. I hope the general tenor of my remarks will show, that my object is truth—not to agree or disagree with any one. Certain it is, I love my profession, and honor its members as the benefactors of my species; and no one of them more than Prof. Bartlett.

The truth is, I look upon his work as an exposition of our knowledge upon the subject; and as it is, or ought to be in the hands of our profession generally, it is convenient as a matter of reference. Valuable as that work assuredly is, I do not think it an objection to the publication of observations made in the West and South, but rather an additional reason for such publications. Differing widely in climate, and in the habits of the people, as this country does, from France and New England, even the same disease may reasonably be expected to present modifications in symptoms, and to require corresponding modifications of treatment; at least observations made in those regions must be verified here, before they can be implicitly relied on. I hope my professional brethren will read this essay, compare the facts and opinions therein contained, with what they have observed in their own practice, and extend to me that charity which a knowledge of the difficulty of properly executing such a task shall suggest—and I hope (may I not be disappointed) that many of them at different points will follow the example, and give me an opportunity of profiting by their labors.

To this essay it may be objected that it is unnecessarily minute in some particulars, or that some portions are even irrelevant to the subject. I feel the objection; but I likewise know that in examining subjects not thoroughly understood, it is difficult to determine the weight which circumstances apparently minute and unimportant may assume; or what may prove relevant or irrelevant to the subject. Certain it is that during my researches since beginning this essay, my views upon some important points have been materially modified.

---

### CHAPTER I.

### ARTICLE 1.

#### NAME.

It frequently happens that a name of disease, which to one person, and in some circumstances, seems peculiarly appropriate, to other persons and in different circumstances, appears very differently.

Such is particularly the case with regard to the term "*Typhoid Fever*," which has been applied to this affection. The term typhoid had had a signification sufficiently definite, as indicating that state of the system, characterised by a quick, weak pulse, a dry, black tongue, great muscular weakness, frequently subsultus tendinum, low delirium, petechia, &c. This signification however has become sadly unsettled by its new alliance. It now has no necessary connection with any one of the group of symptoms, which were formerly necessary to its existence. It is now applied to a state in which the pulse may be strong or weak, quick or slow; subsultus or not, tongue dry or moist, with or without delirium; with or without petechia; occasional epistaxis, a meteoric state of the abdomen; diarrhea.

In fact many cases of typhoid fever, run their course without a single symptom which characterises the "*typhoid state.*" Hence it has happened that two physicians talking of typhoid fever, attached very different ideas to the phrase; one having dothinenteritis in his mind, the other, the typhoid state of bilious fever. I fear that ambiguity arising from this mode of expression, has found its way into our standard writers *eg*. Prof. Bartlett speaking of certain cases, says: "So far from being cases of typhoid fever, even the typhoid state was not present." What meaning does he attach to the phrase here? I apprehend most persons will respond, that state indicated by quick, weak pulse, &c., &c. Yet it seems clearly meant to indicate something necessary to constitute typhoid fever; which as before observed, is not true, of that train of symptoms. On the other hand, to speak of typhoid fever existing without typhoid phenomena, is very close akin to a solicism. It is to be regretted that a phrase of well established meaning, should have been used in a sense materially different from that meaning.

Our phraseology ought to be so uniform, that one man of fair attainments in the profession, should easily understand any other one, when delivering his opinions on medical subjects. I therefore think it unfortunate that the term typhoid fever has been appropriated to this disease. But as the professional writers seem generally to have adopted the phrase, I must consent to the verdict.

It is unfortunate too, that names of disease should ever become fashionable. Some years ago, all cases of fever were congestive—now they are all typhoid fever. I have known cases reported as severe attacks of typhoid fever one day, who were walking the streets the next. I have seen divers forms of disease, febrile, or not, called typhoid fever.

So that the diagnosis is not, in all cases, and to all physicians at least, as clear as noon day.

This disease is called by various names by different writers: as typhoid affection, dothinenteritis, entero-mesenteric fever, follicular enteritis, typhus fever, putrid fever, adynamic fever, ataxic fever, enteric fever, &c.

---

### ARTICLE 2.

### HISTORY.

In the summer of 1841, I was desired to visit a young lady, in whom I took much interest. Her case was quite grave, and it appeared to differ materially from any form of fever, with which I was acquainted.

This difference, I was more able to feel in my judgement, than to describe to others; the principal points being a greater disturbance of the nervous system, and prostration of the muscular power. I availed myself of the assistance of the late Prof. Richardson; but it appeared to me, that the disease was new to him as well as to myself. Two months after this attack, five other members of the same family were seized, a good deal in the same way. It now occurred to me, that the disease was most likely, a form of fever, which had been very prevalent about Paris, Kentucky, several years before, and which as I supposed, had been about Lexington for some years. This I was more disposed to believe, because there had at this time, been a good deal of sickness between the residence of this family and Lexington. Under this view of the circumstances, I felt it my duty to request that some physician, residing in Lexington, might see these cases with me. Dr. Holland was selected by the family, under the belief that he had a very extensive practice, and that he was very successful in the treatment of fever. When he had examined the causes, he gave it as his decided opinion, that it was the typhoid fever of Louis; saying at the same time, that he was very familiar with it, as it appeared about Lexington. Upon my inquiring of him if any dissections had been made about Lexington so as to demonstrate its identity with dothinenteritis, he answered in the negative. Seven cases occurred in that family, two of which were fatal; a third, a lady of about forty-eight or fifty, who was not supposed to be dangerously ill, died instantaneously without a groan; and a fourth, the first patient taken, recovered of the fever only to go into phthisis, of which she died six months after. One other case occurred in that neighborhood about the same time. During the ensuing winter, the disease appeared in our village, and from that time, it has rarely been altogether absent from our neighborhood, although it has at no time raged to as great extent as it appears to have done in many places.

The time just mentioned is the first, as I believe, that I ever saw the disease. I think so, because it appeared to me at that time, to present a phase new to me; and carrying my remembrance back carefully as in my power, I am not able to identify any form of fever with it. In the winter of 1827–'8 I saw a form of fever in Union county, Kentucky, which I thought at one time, might have been the same; but upon referring to my notes of that fever, taken at the time, I am satisfied that the suspicion is untenable. In the winter of 1837, we had a form of fever here, which my friend Dr. Craig thinks now, was the same fever. I am not able to identify the two as one. I recollect very distinctly that purgatives were very prone to run off with watery stools; and that there was considerable disturbance of the nervous system. But I do not remember, that there was much disposition to diarrhea, when no medicine had been taken; neither can I recollect the features of that epidemic, as those most conspicuous in typhoid fever. I have a distinct recollection too, that there was a material difference in the general appearance of the stools. Although thin, they were less watery than those of typhoid fever. They were also of a decidedly green bilious character. There was however, one exception which made a strong impression upon my mind at the time. In that case, the fecal discharges had a very striking resemblance to those at present observed in typhoid fever. Like the latter, too, it went on to recovery, without that change as to consistence and color, which we then considered essential to convalesence.

This fever bore a considerable resemblance to that of 1827–'8 above mentioned. Dr. Gano, too, who has been practicing here upwards of twenty years, thinks that for a long time, he has seen this fever. He describes an eruption as having appeared upon the neck and face, and occasionally upon the arms and body, which corresponds with the rose colored lenticular spots of typhoid fever.

---

### CHAPTER II.

### ARTICLE 1.

#### CAUSES—AGE, SEX, RACE, AND PERIOD OF RESIDENCE.

*Age, recency of residence and contagion* are the circumstances enumerated by Louis and Bartlett as of most importance as causes of typhoid fever. I shall append a table of cases, which I have seen during the last twelve months, which will show age, sex, and color of each patient. It will be seen, that of forty-three cases, twenty-six are between fifteen and thirty years; nine over thirty; and eight under fifteen.

The table shows twenty-seven males, and sixteen females; thirty whites; thirteen blacks. A superficial examination of this table would perhaps convey a very erroneous impression. It will be observed, that of the twenty-nine cases originally mine, eighteen were whites; eleven blacks; of these three whites and no black died.

On the other hand, of the fourteen, whom I saw secondarily, twelve were whites and two blacks; of whom five whites and both blacks died. Consultations are more apt to be held over whites than blacks; therefore the proportion of deaths amongst the whites, in the number seen is unduly large. For the same reason, fewer blacks over whom consultations are held recover. Thus both of those mentioned died. For this reason, I think the cases originally mine, are the proper basis for our estimates. It may be proper to state, that the proportion of whites to blacks, within the bounds of my observations, is about 108 to 100.

I had supposed there was a majority of blacks, but from observations made with some care, though not able to say precisely, I am fully satisfied of the proximity to truth in the statement above made. Our population is stationary to a great degree. The principal ingress is from students to our schools, male and female, and journeymen mechanics

We have about two hundred students from a distance. Among these, I have not known of more than eight or ten cases of fever in the six years; and I do not know that any one of them had been here less than a year. Neither do I know, with the exception of four, that any case was one of typhoid fever,* I presume, however, that some of them were. This table shows a large proportion

*Since this was written, I have seen a number of strangers with the fever, still however, not a sufficient number to think that they are at all more liable than permanent residents.

of cases between fifteen and thirty years—a much larger proportion of cases over forty years than seems to be commonly found—a large majority of males—a large majority of whites—and we may say nothing respecting those of recent residence amongst us. The field is too narrow to found any general conclusions upon. We can only say such has been the result of facts observed by a single individual in a certain location, during a particular year.

TABLE OF CASES REFERRED TO.

| NAMES. | AGE. | SEX. | COLOR. | FATE. |
|---|---|---|---|---|
| J. Stevens | 10 | Male, | White, | Recovered. |
| M. Stevens | 8 | Female, | " | " |
| *M. Daviess | 19 | " | " | " |
| Mrs. McDaniel | 45 | " | " | " |
| Taylor | 15 | Male, | " | " |
| Reuben | 14 | " | Black, | " |
| Elijah | 16 | " | " | " |
| Harriet | 17 | Female, | " | " |
| Sam | 20 | Male, | " | " |
| J. Wallum | 20 | " | White, | " |
| *R. Stubbs | 19 | " | " | " |
| *D. Stubbs | 14 | " | " | " |
| A. Stubbs | 23 | " | " | Died. |
| H. Stubbs | 12 | " | " | Recovered. |
| Frances Stubbs | 17 | Female, | " | Died. |
| Eliz. Stubbs | 12 | " | " | Recovered. |
| J. Hall | 65 | Male, | " | " |
| Miss Conway | 25 | Female, | " | " |
| M. Johnson | 35 | " | " | " |
| L. Bell | 15 | Male, | " | " |
| Caroline | 25 | Female, | Black, | " |
| George | 6 | Male, | " | " |
| *J. Sutphin | 21 | " | White, | Died. |
| Mrs. Sutphin | 50 | Female, | White, | " |
| *Mrs. Brown | 25 | " | " | Recovered. |

| NAMES. | AGE. | SEX. | COLOR. | FATE. |
|---|---|---|---|---|
| Geo. Brown | 30 | Male, | White, | Recovered. |
| *Dick | 19 | " | Black, | Died. |
| Julia | 45 | Female, | " | Recovered. |
| Milly | 45 | " | " | " |
| Lewis | 25 | Male, | " | " |
| *Wm. Buck | 17 | " | White, | Died. |
| *Browning | 25 | " | " | " |
| *Bradford | 25 | " | " | " |
| Johnson | 18 | " | " | " |
| *Burgess | 20 | " | " | Recovered. |
| *L. Flournoy | 25 | " | " | Died. |
| †E. Duncanson | 12 | " | " | Recovered. |
| †Jane | 15 | Female, | Black, | " |
| M. Kershaw | 30 | Male, | White, | " |
| *Will | 30 | " | Black, | Died. |
| *Miss Tapp | 25 | Female, | White, | Recovered. |
| *Mrs. Black | 25 | " | " | " |
| James | 19 | Male, | Black, | " |

I did not see those marked with a * in the first instance, I saw them generally during the third or fourth week; four of them I saw only once; three thrice; the other seven I continued to attend until death or recovery. Of the last number, three died. Of the other seven, four died; being just one half of the whole. Of the twenty-nine whom I saw at the commencement of treatment, three died. This great disproportion is explained by the fact, that the first mentioned fourteen were far advanced, and bad cases at the start. Of the twenty-nine, two were treated by diet and regimen alone.

Those marked † were cases which I had treated in 1843, for the same disease. To enable my readers to give to this circumstance its proper weight, I will add two remarks: 1st. These two are the only certain cases of a recurrence of the disease in the same individual, within my knowledge*; but I do not now recollect any case of fever of any kind, recurring in the same individual during the same period. 2d. This occasional recurrence does not prove that this is not a specific disease, because such recurrences take place occasionally in small pox, and other analogous diseases. Wm. Buck had been here about eighteen months. All the others were permanent residents.

By referring to my cases, I am enabled to make out the following tables. It is to be remarked however, that these tables are not to be considered altogether correct, as many cases are not noted; and it is impossible to say what proportion the omitted cases bear to those noticed at any time, or the sex, or color of such commission.

Of the 192 patients there occurred in

| Dec. | Jan. | Feb. | Mar. | Ap. | May, | June, | July, | Aug. | Sept. | Oct. | Nov. |
|---|---|---|---|---|---|---|---|---|---|---|---|
| 3 | 12 | 16 | 8 | 26 | 21 | 21 | 25 | 26 | 17 | 8 | 9 |

| Total—Winter, | Spring, | Summer, | Autumn, |
|---|---|---|---|
| 31 | 55 | 72 | 34 |

Table of sex and color:

| Male, | Female, | White, | Black, |
|---|---|---|---|
| 104 | 88 | 118 | 74 |

Of the 192 nineteen were forty years old and upwards.

I do not know that I appreciate properly the title of a recent residence as a cause of disease. If we consider typhoid fever as a specific contagious disease, to which a person is not usually liable a second time, we will not of course suppose that a person after having it once will, by changing his residence renew his liability. I presume that the activity of this cause is adventitious rather than

---

*During the present year, 1850, I have seen two cases which I consider well marked, occurring in persons who had previously suffered an attack. I have also seen some cases, which occurred in persons who were reported to have had the disease previously, and who probably had, but as I did not see them in the first attack, I do not think myself justified in stating them as cases of a repetition of the disease.

necessary. I suppose that a large portion of those persons who flock to large cities are young persons, precisely within that period of life, at which the liability to fever of all kinds is greatest—that the change in the mode of living is considerable—that there is an unwonted and undue excitation of the various passions—which all acting simultaneously, have a very great tendency to disturb the regular healthy play of the functions, and of course to produce fever. For this reason I presume this cause is less influential in our rural districts.

---

### ARTICLE 2.

### DWELLINGS, ETC.

In every case coming under my observation, where a considerable number of cases have occurred in the same family, there has been reason to think that something connected with the situation of the house, may have contributed to the prevalence of the disease.

For instance, at Stubbs', the house was old, and dilapidated; the windows in the upper story open, so that there was free admission of wind, rain, and snow; the cellar, which had usually been occupied as a kitchen and dormitory for the negroes, had during the winter, when the disease prevailed, been closed, and made the receptacle of all kinds of filth, into which the water from the house and yard found its way, for want of proper care to drain it off. At John Laws', where the family suffered severely in 1845, the house was in very much the same situation, except there was no cellar. At all other places, where there has been much sickness, the houses are without cellars, and without means of admitting air freely beneath the floors. I have had the floor taken up to examine the state beneath, but have found no sufficient cause.

It is proper to add, that this condition is almost universal among the negro houses, and also among such dwellings for whites as have remained from the early settlement of the country. It is also proper to state, that although the statement above made is true as to those families most afflicted, yet the disease has by no means been confined to such houses, but has been found in new houses of the most comfortable construction.

Very lately I have seen a number of cases which may be thought interesting. On the 3d of April, Mrs. C., about twenty-two years old, became ill of typhoid fever, I saw her on the 10th—then no fever known any where in the neigeborhood. She had come home, after an absence of two or three months, some time in February—no fever known in the neighborhood from which she came. The house in which she resided is an old brick, built in 1794, but in a good state of repair and clean. On the 19th Isabella, the woman who waited in the house, sickened. May 1, Moses, Isabella's brother, sickened—he slept in the same house, but not in the same room with his sister—also a negro man thirty years old. About the same time a boy two years old was attacked—this child slept in another house. During the next week two sisters of this boy, sleeping in the same house, were seized, as also a negro girl, nine years old, and daughter to Isabella, and spending the day in the family dwelling, and the nights with her mother. Also a negro man who slept in the kitchen. May 10th, Mr. C., husband to the lady first sick, and his mother, became ill. About this time, May 20th, a brother of Mr. C. came from an adjoining county, and had some chip rubbish removed from the immediate vicinity of the family dwelling. He spent two or three days at the house, returned home, and was taken sick in ten or twelve days. Another brother visited the family two days after the return of the first: upon his return home he had a severe attack. At the time of this last visit, a brother-in-law and sister with their children and a negro woman as a nurse, arrived on a visit from Missouri. On the 13th of June the sister and nurse were attacked—on the 28th and 30th two of the children became considerably indisposed, remained for two weeks with what seemed to be a mild attack of typhoid fever. A negro man who spent two days there and assisted in laying out a negro who had died sickened on the 16th of June. And my son who had visited the family daily and spent a week, night and day in the family, during their greatest distress from the 16th to the 23d of May, sickened at the end of that time.

A peculiar fatality seemed to attend this spot, as every member of the family complained more or less; and of twenty belonging to the family, eleven had serious attacks, not counting the visitors. The houses in which the negroes were sick had the floors nearly on the ground with no chance for ventilation under them. One house afforded sufficient means for ventilating the rooms inhabited. In the other, consisting of two rooms, one about fifteen feet square, the other ten, circumstances were not so favorable—in the large room were two beds with curtains, a bureau, table, a chest for clothes, and several chairs, besides a truckle bed under one of the others; this room has an outer door, a small window, and fireplace. In the smaller, was a bed, chest, and chairs—no outer door, one very small window near the door opening into the larger room, no fireplace—the ceiling six feet high—both closely chinked and daubed. At the corner of the kitchen, about twenty yards south of the door of the dwelling house most used, and about the same distance east of the negro houses, was a slop trough, which leaked badly, and suffered the slop to accumulate in a sort of mudhole beneath, giving rise to a very offensive smell. These houses are situated on a bluff some forty or fifty feet high, with a large cluster of trees between them, and a creek which runs on the east and north, which however, had not been sufficiently low at the outbreak of the fever to be suspected as a source of disease.

The sickness was almost entirely confined to those persons who spent their time at and about the dwellings, the field hands being less affected. It is proper to observe that for two weeks during Mrs. C.'s illness, her mother remained with her continually—her father and several of her brothers were there much of the time, but none of them became affected with the disease.

I may remark that this family lives about half a mile from Downing's, and three quarters of a mile from Mrs. Lightburn's, hereafter to be mentioned.

In a considerable proportion of cases, it has appeared to me that the attack was brought on by an undue exposure to cold.

---

### ARTICLE 3.

### CONTAGION

Is considered by many as a potent cause of this disease, I shall therefore adduce some facts as observed by myself, and some which have been detailed to me by others in whose accuracy of observation I have full confidence.

1st. Willim Stubbs sickened in Georgetown during the third week of December, 1845, and removed to his father's about three quarters of a mile from town, his father's family being then in good health. This too was about the time when the disease appeared in town. After an illness of three or four weeks he died. About two weeks after his death, two brothers were seized with the fever. About the time they could be considered convalescent, two other brothers and two sisters were confined. One brother and one sister died, and while the other brother and sister were still sick, a young lady, resident in the family sickened. And about two weeks after, the mother sickened, and at the expiration of three weeks died. Thus in a family consisting originally of eleven, all whites, nine were sick and four died.

2d. In May, 1846, John Sutphin visited his sister at Leesburg, who at the time was ill of the fever; he staid with her for several days, and shortly after his return he sickened. About three weeks after his attack, his sister residing in the house sickened. A few days after, a negro boy was taken. In about a week again his mother and a negro woman, mother to the boy, were taken. About two weeks more, a brother-in-law, husband to the sister was sized. Of these, John Sutphin, his mother, and the negro boy died. In the mean time the physician first in attendance on the family sickened, and eventually died. His student, who had been a good deal at Sutphin's, and also waited on him, sickened and eventually died.

3d. Young Withers came home indisposed, continued to get worse, and after an illness of four or five weeks died. Four brothers and sisters sickened at intervals of four or five weeks. All represented to have been cases of typhoid fever by Dr. Emison.

4th. On the 23d of May, 1841, Rachel Cooper was taken—the fever then prevailing in a family three miles off—whether she had visited that family is not known. On the 26th of July, I visited her mother and four negroes, who had all been somewhat indisposed for several days. On the 22d August, Jane, daughter to one of the negroes, and sister to the others was taken. So, in divers families the disease has attacked different members after shorter or longer intervals.

5th. The disease appeared at the Stamping Ground, nine miles from this place, about the end of December, 1845. At Christmas, Miss Sinclair left Georgetown indisposed, and sickened upon her arrival at the Stamping Ground. It is said that most of the first cases in that neighborhood had visited her on her return.—*Dr. Black.* I have it however from Maj. McCalla, a relative, and whose connexions about the place were very intimate, that the first cases which occurred in that vicinity *had not* visited Miss Sinclair. And that himself, who had a severe attack, had not seen any one ill of the fever.

On the other hand, whilst a majority of *cases* are found in clusters, a majority of *families* in which the disease appeared have only one or two sick—at least within a range of time during which contagion can be relied upon as the cause.

1st. During the winter of 1845–'6, some twenty or thirty families of our village were visited by the disease; I think two was the greatest number attacked in any one family. It is true that during the last summer, 1846, some of these families had a case.

2d. At this visitation, the disease appeared in different parts of the town, and in families which had no communication with each other. Miss Sinclair, who was supposed to have carried the disease to the Stamping Ground, was an inmate of Thornton F. Johnson's boarding school, the young ladies of which do not visit among the citizens; and there had been no case of sickness in the family. Again, Wm. Stubbs boarded in a family in which no one had been sick; neither had he seen any one sick since September, when he was said to have visited John Young. I do not know of what disease Young died. Stubbs sickened on 24th of December, after exposure to severe cold. There had previously been a few cases scattered about town, and about this time it became quite common.

Again, on the 1st of August, 1846, I was called to visit five patients at Mrs. Lightburn's, three miles south of Georgetown, all of whom had been attacked within a week. One of these cases was so mild and indistinctly marked, that I did not consider it as a case of fever. At this time there was no sickness of any kind in that neighborhood; these negroes declared that they had not seen any body sick with any disease. It is true that on the 11th of January, preceding, a boy of twelve years old had the disease in the same family. It is also true that on 22d of September, 1845, four months before this last, I was called to five negroes, one woman and four children, (who were all seized about the same time,) at John Downing's, who lives about half a mile from Mrs. Lightburn's. I think it material to state these facts, and also that no communication was known to have taken place between the negroes at Mrs. Lightburn's and those of Mr. Downing.

The cases at Downing's also were without any clue to connect them with antecedent cases. These cases are all that occurred in that neighborhood, so far as I know and believe, from 1841 to December 1846.

3d. On the 22d of December, 1845, I visited an apprentice of Tyson Bell, who had been ill several days. On the 24th I prescribed for his bed-fellow of about the same age—he however walked the same day seven miles; was confined next day, and had a severe attack. Two journeymen occupied the same room, and one of them at least was a stranger, but neither contracted the disease. Again, during the summer of 1846, a negro woman had the fever, and although very ill, she was compelled to attend to her child six months old, during all of her illness—the child escaped.

I have now given some of the most forcible examples which have come under my observation, tending to show the contagiousness of the fever; and others to militate against its contagiousness. Let us examine them and see to what conclusion we shall arrive. It is easy to understand how a physician with his view fixed upon the first list of cases, should be entirely satisfied that the disease was contagious; nor is it difficult to perceive how one, who should look at the other list, should come to a conclusion altogether different.

It is not necessary that I should read a homily on the difficulties surrounding this subject, contagion, in promoting the spread of disease. Wiser heads than mine have felt the weight of those difficulties, and abler pens have portrayed them. Neither ought we to be too confident that the laws of contagion are at present fully understood. For instance, one of the laws of contagion, as at present interpreted, is that the time between the infection and that of the appearance of the disease "is scarcely ever less than seven, or more than twenty-one days.—*Parr in Voce.* "One, two, three, or more days, and in some cases though much more rarely, even for weeks after."—*Wilson on febrile diseases.* 1*st Amer. Ed., Vol.* 1, *p.* 181. Now it has occurred during the last summer, that a young lady who at the time was a room-mate with three others laboring under small pox, she herself having been vaccinated, saw and played with a child on the 18th of May. The young lady broke out with the varioloid on the 12th of June, and the child with the small pox on the 17th July, (being sixty days from the time she was with the young lady mentioned;) having had no other known chance to receive the infection.*

---

*I am admonished that there is probably some mistake about this case. I can only say I have made the statement on the authority of Dr. Hood who attended this case and others which grew out of it—that he was entirely satisfied as to the facts—that at the time the young lady played with the child, small pox existed in no house in the county except the house in which she lived and had not been recognized there as small pox—that upon its recognition, June 1st, great alarm pervaded the country. That it appeared in only three families out of town, and

Again, the distance at which contagion is efficient "is certainly not above a few yards, probably not above a few feet."—*Wilson, v.* 1, *p.* 178.

Another difficulty in our way, is the almost impossibility which frequently exists of arriving at all the facts of the case. Many persons will run risks of contracting disease, and manfully deny those risks. Yet if they escape, they will afterwards brag of their fearlessness. In our country this is especially true of the black population. No faith can be placed on their asseverations. However, this may be explained, I am every way satisfied that it is a fact.

Again, we are told that certain diseases although usually spreading by contagion, do yet occasionally originate spontaneously. "Whatever be the difficulty of tracing the source of other contagious diseases, it is no difficult matter to detect that of typhus. The combination of a very few circumstances, and those of frequent occurrence, is sufficient to produce the complaint. Typhus, that is, fever in which debility prevails, may arise in ill ventilated and crowded places."—*Wilson, v.* 1, *p.* 175.

"According to the observations of M. Gendron, typhoid fever propagates itself very slowly by contagion. The interval between the successive cases varies from three weeks to a month; so that the fever is often several months in spreading through a village or neighborhood. The period of incubation he thinks rarely exceeds eight or ten days, though it sometimes extends to fifteen, and is occasionally as short as twenty-four hours.

"He is also led to the conclusion that the power of

---

they widely separated—that that in which the child lived was farthest from town, and had no communication with any infected family except that stated in the text.

This is another example of the uncertainty of spontaneous origin of contagious diseases.

transmission, or communication does not exist in the early period of the disease; that it is rarely active before the sixteenth day; and in general terms, that it continues from the third week for an indefinite period, including convalescence. He states some facts which seem to show that the contagious matter may remain in a bed for two or three years."—*Bartlett on Typhoid Fever, p.* 82.

Again, "it is easy to see that this question is one of great practical importance. It can be fully settled only by further and more various observations; and these observations for obvious reasons, can be best made amongst the scattered population, and in the small villages in the country." *Id. Loc. Cit.*

Having stated some preliminary matters, let us now turn to the subject in hand. The chain of cases as they occurred in the family of Stubbs, Sutphin, Withers, and others which might be mentioned, was very much the precise course which is observed in families afflicted by the small pox, measles, scarlet fever, and other diseases, acknowledged on all hands to be contagious. Therefore, contagion is capable of producing such a chain of events. But it is equally true, that the same course is frequently observed in cases of bilious, intermittent, and remittent fever.

I have many times seen one member of a family after another confined with this last form of disease, so as to give an apparent reason to believe in its contagiousness.

Again, I have seen a whole family so afflicted, that there were not enough well to wait upon the sick. We have a right to conclude then, either that bilious fever is contagious or that typhoid fever *may* not be so.

As several cases appearing successively in one family do not prove conclusively that such cases are kept up by contagion, so a single case appearing in divers families does not prove that such cases are not of a contagious character. I can readily believe that such facts take place in scarlet fever, measles, &c., though I do not now recollect any instance of it.

But it is said that diseases which are usually propagated by contagion, occasionally originate spontaneously. I apprehend however, that this is more frequently true in appearance than in reality.* A person may receive an infection and not be conscious of having been exposed to contagion, and the disease appearing afterwards, it is supposed to have arisen spontaneously. This circumstance has transpired twice in the last three years in this town. On the 9th of April, 1844, a young lady arrived in our town; on the 21st she broke out with measles. At that time we had no measles in our town; but we very soon had plenty. She was positive that no measles existed in the neighborhood from which she came, and that she had seen no person with it whilst on the journey. This version is the more worthy of credit, because she was very apprehensive upon the subject; having been strongly impressed by her family physician that measles would be to her particularly dangerous.

Again, in the latter days of April, 1846, the small pox appeared here, in a room occupied by three young ladies, each of whom took it one after another. Great difficulty existed in discovering the manner of its introduction. At length it was ascertained that a fourth young lady, who had been vaccinated, had arrived at the house two or three weeks before the disease appeared, and had been much in company with these ladies; and further that she

---

*I certainly do not mean to say that contagious diseases *never* originate spontaneously. In a practice of thirty years I have seen one case of scarlatina which seemed to arise spontaneously—but one, as at present recollected. The object of my argument is to impress my conviction that typhoid fever arises spontaneously *too frequently* to admit of its being classed among contagious diseases.

had been confined two or three days by indisposition during her journey; and that she had, on her arrival here, two days after her confinement, an eruption, the character of which, as it attracted no particular attention at the time, could not be ascertained. Years before, she had seen small pox, and expressed her opinion that the young ladies above mentioned had that disease. She had not recently seen any one with the small pox, and had no suspicion that she had herself undergone an attack of varioloid, yet such doubtless was the fact.

But according to Sir Wilson Philip, or Philip Wilson, there is no difficulty in detecting the source of typhus contagion—"few circumstances, and those of frequent occurrence are sufficient; ill ventilated and crowded places are all that is necessary." I however do not believe that those circumstances do occur in this section at all.* The dwellings of our population both white and black, are generally sufficiently ventilated. They consist for the most part of houses but a single room deep, sometimes two, into each of which entrance is through a door in the outer wall, and usually a middle door. There is always a chimney, and usually a window or two. In one of these rooms, say of twelve to eighteen feet square, a family of negroes, say of five to eight, frequently sleep. They usually, even in the summer time, have more or less fire in the chimney every evening. This is the best show for ill ventilated and crowded places which we can furnish in this region. When we reflect that these chimneys very frequently would admit a flour barrel readily, and the fireplace appears to have been constructed more with a view to the destruction than to the saving of fuel, the dwellings too, composed of logs, rarely closely filled in be-

*One of the negro houses at Mrs. C.'s before mentioned comes nearer the description than any house I have seen.

tween; I really do not think that such habitations can be called ill ventilated.

When a contagious disease first makes its appearance in a neighborhood, it usually appears not only in a single house, but in a single individual, or it may be in two or three, in quick succession, if so many have been exposed to it at once. It rarely or never appears about the same time in different families, in the same neighborhood, those families having little or no communication with each other. Yet such was its mode of access in Georgetown in the fall of 1845; and such, if we may believe Maj. McCalla, its advent upon the Stamping Ground, the ensuing winter. Again, five cases occurred at Downing's in the fall of 1845; these were in a negro woman and her four children. These were all seized almost simultaneously; they occupied a single room, and no disease was known in the neighborhood.

At Mrs. Lightburn's four cases ocurred in August, 1846, almost simultaneously—these occupied three rooms.

At Mr. Cooper's in 1841, five cases occurred in a like extremely short period, the mistress, a negro woman and her three children. In that family, the negro woman had been little exposed to danger from the case which had occurred in the family two months previously, the others not at all—these four negroes occupied three rooms.

It does appear to me that these cases are altogether irreconcilable with the idea of contagion. No disease being in the family, or indeed in the neighborhood, four or five persons are attacked in a family by a disease (which spreads slowly by contagion) within the space of two or three days. If contagious at all, these, (at least two of them,) must have been cases of spontaneous origin because there was no source to which the disease could be traced; and besides, too many cases occurred at once, to suppose that the disease had been introduced from other families. But it would be very strange, that circumstances sufficient to produce an outbreak of a contagious disease, should exist, and yet that the disease should spread no farther after those first, and simultaneously attacked. This, however, was the case at Mr. Downing's and Mrs. Lightburn's.

Take another case: Tyson Bell's two apprentices sickened, with an interval of about a week. Two roommates, of an age considered particularly favorable for the invasion of typhoid fever, and one at least, if not both of recent residence in our town, escaped. Again, during the fall of 1846, a negro woman was seriously sick at the same house, and was compelled to attend to her child during the greater part of her illness, but the child escaped. In addition to this, I have known many cases where the patient was one, whose situation was calculated to produce the greatest solicitude, and of course the members of the family were much exposed to danger, if the disease is contagious, who nevertheless all escaped. I have in my mind now a lady of twenty-five, married ten years, with no children, but extremely anxious to have some, in the eighth month of pregnancy, and having numerous relatives; during winter sick for four weeks, and eventually dying without communicating the disease to any one of her numerous friends.

From eight to fifteen days is the time of incubation as allowed by Bartlett; but I have already expressed an opinion that we have not all the information on that point which is desirable. I have given a case too, in which it seems well ascertained, that the infection of small pox remained dormant about sixty days. It is true, that contagious matter in the state of fomites, may remain in a state fit to become active, for an indefinite length of time. Bearing these facts in mind, and allowing them all due weight, it does seem to me that it is still too much to ask of contagion an explanation of the cases at Downing's and Lightburn's.

At the time the cases occurred at Downing's in 1845, I am not aware that the disease was any where in the country, certainly not in that neighborhood.

Four months afterwards, it appears in a boy not known to have visited Downing's, and not likely to have done so, being rather too young to run about the neighborhood. Again, seven months after this, four cases occur at once in three different houses, one of them it is true, in the same house as that in which the first was sick. This was the dwelling house of the family, plastered and painted, and altogether as unlikely to afford a lurking place for fomites as any other house. But fomites here could not affect the other three, as they did not go into the house, being field hands. I have said that no reliance can be placed on what negroes say, as to their visiting sick acquaintances. But their denial of such visits does not prove that such visits were made. When there are no sick to visit, they may be believed; and further it is fair to state that those sick at Downing's were precisely those least likely to visit, if there had been cases in the neighborhood, viz.: a woman and her children, the oldest child twelve years old.

According to my idea of a contagious disease, the presence of contagious matter is, with very rare occasional exceptions, *essential* to its propagation. It is not necessary that every person exposed to the contagion shall become infected. On the other hand, circumstances may exist which shall prevent to a great extent, or even altogether, the spread of the disease. Seed may be sown in the earth, and afterwards so damaged that germination will not ensue; yet no germination can take place where there is no grain to germinate. A contagious disease, whose infection may remain dormant in the system for seven months, or be quickened into life in as many hours; which may appear in half a dozen members of a family, in as many days, without any chance of introduction from without, and then spread no farther in that family, or neighborhood—which can originate in divers families in the same village or neighborhood, those families having no communication with each other—which can originate frequently at divers points in the country, and infect but one or a few persons—which, in one word, shall frequently set at nought all the laws of contagion as usually understood, although it may at times seem to obey those laws, is a contagious disease which I do not profess to understand. Neither does it seem to me, that anything is gained in etiology, by calling in the aid of such an agent.

---

### CHAPTER III.

### ARTICLE 1.

#### SYMPTOMS—MODE OF ACCESS.

In a considerable proportion of cases, the attack comes on suddenly with a marked chill; and occasionally light chills are repeated at irregular intervals.

Most commonly however, the patient complains of indisposition, accompanied by muscular debility, and disinclination to mental exertion. In these cases, the alteration from day to day is so slight that the patient frequently becomes decidedly ill before he is willing to acknowledge that there is anything the matter. Many cases which come on thus gradually, remain mild throughout; others however increase in severity continually, and become the very worst cases of disease. In one case, to be mentioned hereafter, the attack seemed to be ushered in by a state of congestion. I have seen two cases in which the attack commenced with inflammation, swelling and hardness of the breast so as to threaten mammary abscess. Also several in which a dysenteric state of the bowels existed for a day or too; when the disease assumed its usual form.

---

ARTICLE 2.

PHYSIOGNOMY AND NERVOUS SYSTEM—EXPRESSION OF COUNTENANCE.

SECTION 1. When called to patients laboring under this disease, we are frequently surprised to see the great placidity of countenance. This is so marked in many instances, that we might suppose there was nothing the matter.

This placidity frequently continues throughout the disease to convalescence or death. In many cases, however, there is in the countenance, a very marked indication of suffering. In some of these cases, the countenance loses its expression of suffering in the progress of the disease, without there being necessarily any abatement in its severity.

SEC. 2. The *sensations* correspond very much with the expression of countenance. When this is placid, the patient replies that he is "right smart," pretty well," "better to day," or something of like import. On the contrary, when the countenance is indicative of suffering, he complains of his head, his back, his limbs, of muscular soreness, or some indefinite sensation of uneasiness.

SEC. 3. *The state of mind* seems to be that of weakness rather than of perversion. In a good many instances, although there appears no perceptible affection of the mind, there is a total forgetfulness of everything which occurred during the attack. I am of the opinion that this is true much more frequently than is supposed. A few days ago, I saw a lady whom I attended in 1844, in an attack of medium severity, and whom I did not at any time, suspect to be at all affected in her mind, neither did her attendants. I asked her how much she remembered of circumstances which occurred during her illness? She answered that the first she recollected, was my telling her that I did not think it necessary to visit her any more. The patient does not answer questions readily, and has not a distinct recollection of events, which have recently transpired, as connected with his situation. But the forgetfulness does not extend to events which transpired before his illness. As the disease advances, this state of mind becomes more marked; and anon, aberration of mind is observed upon first waking. To this succeed delirium, especially at night, and low muttering. Instead of this low muttering delirium, in a few cases it is of a furious character, such as may well be denominated raving mania. There is usually more or less headache, especially in the anterior stage of the disease. After a few days, this is less complained of, whether the disease is progressing favorably or not. If progressing unfavorably, shaking the head will occasion pain when none is perceived when it is still.

SEC. 4. *Senses.*—Feeling is rarely much deranged. Sometimes there is muscular soreness which does not admit the limbs to be handled without some pain. *Sight* is exalted; the eyes are not able to bear the usual amount of light, and the patient frequently lies with them closed. *Hearing* is also more acute. Noises which are scarcely perceived by others, are annoying, such as ordinary conversation, or crackling of newspapers in turning them to read. *Tinnitus aurium* is frequently present. As the disease advances the hearing becomes less acute, and occasionally decided deafness takes place. I have made no remarks as to the sense of smell. The *taste* is more or less deranged. How much of that derangement is attributable to the condition of the mucous membrane of the mouth, I am not prepared to say.

SEC. 5. *Wakefulness.*—At the commencement of the disease there is frequently no sleep for several days; and the patient earnestly desires something to induce sleep. As the disease advances, this state may continue, or be exchanged for more or less somnolence, with a somewhat heavy respiration.

SEC. 6. *Muscles.*—One of the most distinguishing features of this disease is muscular weakness. A person may lie in bed, and seem to be but little indisposed, may feel well, but cannot walk or even sit up without an unaccountable sense of weakness, frequently attended by a giddiness and indistinctness of vision. This debility is sometimes so great, that the patient can scarely raise his head. I have seen one case at least in which this extreme prostration was attended by rigidity of the muscles generally. Like all other symptoms, this is not always equally well marked. I have seen a patient sit up on the side of the bed and wash her face and hands within a few hours of death; and turn herself on the side in *articulo mortis.* In the latter stage of severe cases subsultus tendinum takes place.

---

### ARTICLE 3.

### SANGUIFEROUS SYSTEM—PULSE.

SECTION 1. In the commencement of the disease, after the chill, if there has been one, there is usually some increase of force and frequency of pulse, but it is not very marked. I have rarely seen it such as to suggest the propriety of venesection. In a majority of cases, the pulse is from five to fifteen beats more frequent in the afternoon than in the morning.

As the disease progresses, the pulse increases in frequency, but usually diminishes in fullness, until at the acme, it ranges from one hundred and ten to one hundred and thirty, sometimes much higher. I have seen cases again, during which the pulse was little affected during the whole course. Again, I have seen one case at least, in which, the pulse remained at fifty throughout; it was full and laboring and seemed to demand the lancet. In a considerable number of cases, when improvement had commenced, the pulse has fallen in frequency rapidly, and has been attended with the same impression of fullness, and laboring.

Sec. 2. *Epistaxis* I have rarely seen, and it has usually been in persons who are habitually liable to it. Indeed of five persons in one family, which was strongly disposed to epistaxis, or rather in which it was very common, but one is noted to have had it.

During the present year, 1847, epistaxis has been much more frequent in proportion to the number of cases than I have noticed heretofore. One half of all cases having been marked by it. The same remark, however, applies to the measles of the present year. In my own mind, I draw a distinction between epistaxis as it occurs in the early and late stage. In the first instance, so far as my observation has extended, there is a stillicidium of a few drops, of no obvious import, and followed by no appreciable consequences. In the second, the hemorrhage is occasionally alarming, and may be fatal.

Sec. 3. Hemorrhage from the bowels, has been somewhat more common than from the nose. That too, has always been in the advanced stages of the disease, and the cases have been of considerable gravity. I have seen one case, in which there was a considerable discharge of bloody urine. That too was a grave case.

Sec. 4. *Congestion.*—In September, 1845, I saw a case in a middle aged gentleman of Fayette county, who, from the description of his physician, would seem to have labored for four days under symptoms of a decidedly congestive character, viz.: cold, shriveled surface, frequent, small pulse, hurried, panting respiration, attended by sensations of great heat, and oppression in the chest. This state had been removed when I first saw him, it was said, by sponging the body with cold water. After this, the course of disease was that of a severe case of typhoid fever.

---

### ARTICLE 4.

### DIGESTIVE SYSTEM—MOUTH AND TONGUE.

Section 1. At the commencement of the disease, the tongue is usually moist, whitish, with red papillæ sticking up through the white coat—at other times, there is little or no alteration from health, in its appearance. Frequently it is broad, sometimes rather narrow. At this period there is nothing remarkable about the lips. As the disease progresses, the tongue becomes less moist, or at least there is more clamminess about it; the papillæ are more distinctly marked, the edges begin to become red, the taste is vitiated, the lips begin to become red, and somewhat dry. There is frequently a slight soreness of throat, perhaps only observed upon swallowing; and by inspecting the fauces, a slight redness may be observed.

As the fever advances, the tongue becomes more and more dry, sticky, and dark; the teeth become encrusted with sordes, the lips dry and disposed to crack. Occasionally the first coat will leave the tongue red, shining, slick, and dry; sometimes moist, but yet shining. Sometimes the tongue is covered with a layer, very much resembling tubercular matter; at other times, with what might seem to be fine particles of coagulable milk, easily scraped off, but soon reappearing. In a few instances the tongue is considerably swelled; it may be at the same time moist. As death approaches the tongue becomes more and more dry and thick, so much so, that it is difficult to keep it moist enough by giving the patient fluids, to enable him to speak distinctly. Presently he becomes unable to protrude it.

SEC. 2. *Nausea and Vomiting.*—There is rarely nausea or vomiting. Yet in a few instances one or both may be present. But the appetite is gone, or at least there is no disposition to eat. Thirst is frequently urgent. In a few rare instances it happens that the appetite, at least a sensation which the patient regards as an appetite, remains throughout the attack, even until within a few hours of death. In a few instances I have seen towards the close of bad cases, a continued vomiting of dark green bile, very much as in bad cases of bilious fever. Such cases have been attended by yellowish tongue and bitter taste in the mouth.

SEC. 3. *Diarrhea.*—Patients frequently suffer with diarrhea for several days before they are confined to bed. This may consist of frequent small discharges of liquid feces; or there may not be more than one or two dejections a day, but those very large and serous, or they may be only semi-liquid. Again, at the first, there may be no diarrhea; there may even exist costiveness, but this is by no means usual. If the bowels have been costive at first, they frequently are affected with diarrhea after a few days, which indeed many times seems to be brought on by the administration of an emetic or cathartic; but certainly many times without either. These dejections may be semi-liquid, or very watery, of different colors, whitish, greenish—most commonly of the color and consistence of new cider. In a majority of cases, perhaps, not indicating either a deficiency or derangement of the biliary secretion. At least there would appear to be as much bile as is found in dejections of the same degree of fluidity, occurring to a healthy person, if such expression is allowable. These discharges after a few days' continuance of the fever, acquire a very offensive, cadaverous fetor—indeed, at one time I considered this odor peculiar to typhoid fever; but I am now satisfied that I have observed it in other diseases.

Dr. Black, of the Stamping Ground, informs me that he has uniformly seen a deposit of a dark fine powder in these dejections, even before any medicine had been given; I have commonly seen this sediment, but I have always considered it as the black oxide of mercury. I do not think I have ever seen this sediment when no mercury had been taken. I have certainly seen it when none had been taken for several days. I have also seen it frequently in bilious fever, when calomel produced watery stools. It is of course only to be seen when the stools are watery.

For the purpose of determining whether the black sandy looking matter, found in watery stools, is the black oxide of mercury, I obtained some on the 14th May, 1847, from a patient who had taken calomel and blue mass the previous night; I folded the deposite in paper and examined it the next day. Upon opening it I was surprised to see that it had lost much of its blackness; being in fact of a dirty brownish color. It had an evidently gritty feeling between the finger and thumb; when subjected to a microscope of about sixty diameters, it appeared again nearly black, in grains about as large as blasting powder does to the naked eye, and of very irregular shapes. Muriatic acid dropped upon it converted a very minute portion of it into a whitish powder, (calomel I presume,) but the great bulk remained perfectly unaltered by the acid.

Laid upon the point of a spatula and subjected to the flame of a candle no change took place—no odor was emitted. The grains broke upon being pressed on a tyle, like rather soft sand. Having wasted, in these experiments, what little I had obtained I of course ceased for the present.

As a means of ascertaining the probability of calomel being converted into the black oxide in the bowels, I took some calomel, and subjected it to diluted aqua ammonia of different degrees of strength. The articles were those which I had in the shop at the time, the calomel being hydro-sublimed. By dropping the solution on the calomel, the intensity of the black color produced, diminished progressively from one part of aqua ammonia to four of rain water at which I commenced, to one part in five hundred and twelve, at which it became of an ash color. But calomel put into a solution of $\frac{1}{1024}$, and shaken gave a decided color much darker than hydrarg. cum creta. Put into a solution of $\frac{1}{4096}$ it became of an ash color. The oxide, reduced by all these processes, was as might have been expected an impalpable powder, exhibiting no appreciable bulk under the microscope.

This experiment has proved to me that although the black oxide of mercury may be found in the bowels, this particular deposit is not it.

The diarrhea continues more or less strongly marked, generally until convalescence is perceptible. After some days, we have occasionally a few points of pus or mucus, sometimes tinged with blood, and sometimes not. Occasionally a case occurs, in which there is a large quantity of mucus unmixed apparently, with anything else. Sometimes the stools are more consistent and of a dark green color, they differ however from the *bottle green* stools produced in bilious fever, by being of a brighter color and more glossy appearance.

As improvement takes place, the dejections become very gradually of more consistence, and very soon there are observed very small particles of well elaborated feces, of about the natural consistence, mixed with the yet liquid stool. The dejections may become less frequent and of greater consistence however, without there being any improvement of the patient. It occasionally happens there is but one liquid stool a day, and it may be that an injection is necessary to produce that. It may be that after the continuance of diarrhea, for a considerable time, the bowels become costive rather suddenly. I saw a case of the kind last summer, and when we moved the bowels by injection, the feces were of the consistence of health, and of a color which would have been produced by having eaten heartily of stewed dried peaches.

During the continuance of the fever, it occasionlly happens that a worm is discharged from the bowels, and as children are very apt to pick their noses and lips, it is sometimes supposed they are troubled with worms; and vermifuges are given for their expulsion; rarely with any success. There is however no reason why children infested with worms may not be attacked with the fever; and it sometimes happens that considerable quantities are eliminated.

Towards the latter stages, considerable quantities of blood, are sometimes evacuated by the bowels. It is a little singular, that, according to my experience, this symptom is confined very much to particular families. Thus in one family in which there were two cases, it occurred in both. In another, where there were nine cases, it occurred in four. Besides these six cases, I do not think that during the last six years, I have seen more than two or three instances of it.

SEC. 4. *State of Abdomen.*—Connected with the diarrhea I will make a few remarks as to the abdomen, as observed externally. There is tenderness upon pressure usually observed at the epigastrium, and especially at the right iliac fossa, there is very commonly more or less distension and tympanites of the abdomen. This tympanitic condition is not at all removed by the evacuation from the bowels, even when, as sometimes happens, there are enormous quantities of flatus discharged with the feces. On the contrary, the distension is, in many cases, in direct proportion to the amount of diarrhea, increasing as that increases, subsiding as that subsides. There is however no necessary connection between them, as in some cases there is much distension and little diarrhea, and in others considerable diarrhea and little distension. So there may be tenderness without distension, or distension without tenderness of abdomen. Again, there may be a rigidity of the abdominal muscles, without either tympanitic distension or tenderness, or various combinations of these states. Upon pressure at the right iliac fossa, quickly and suddenly repeated and withdrawn, a gurgling noise is often evident, occasioned by the presence of air and fluid in the bowels at that point. I have observed it before diarrhea had been noticed, but that made its appearance soon afterwards.

---

### ARTICLE 5.

### OF THE URINE

There is not usually any marked affection. Frequently it is rather scant. In a very few cases I have observed a suspension for a day or two, and occasionally it is highly colored and turbid. I have lately seen one case in which no urine was discharged for six weeks, except by introducing the catheter. I have seen one case, in which it was mixed to a very considerable degree with blood; and another in which stranguary occasioned by the application of a blister, gave rise to the expulsion of bloody urine with a coagulum as large as my little finger, and an inch and a half long. Stranguary in a very few cases comes on spontaneously.

---

### ARTICLE 6.

### STATE OF THE SKIN.

SECTION 1. *Heat*, *Dryness*, *&c.*—During the first few days of the disease, there is usually an evident increase of temperature, especially of an evening, at which times the face, sometimes both cheeks, sometimes only one, is more or less flushed. This redness of one cheek, shifting over to the other is noted by Dr. N. Smith as a symptom of the disease. I do not remember to have noticed its being given as a symptom of any other disease. It certainly appears in typhoid fever; but I have seen it much more frequently in bilious fever, and especially in bilious pleurisy. It was a very general, almost universal symptom in those diseases from October to March, in that part of southern Kentucky in which I lived, from 1820 to 1833: when there was much febrile excitement, both cheeks were generally, not always flushed. There was no necessary connection between arterial excitement, and the flushing of one cheek. On the contrary it seemed particularly whimsical in its appearance. Sometimes one cheek would be intensely red, even to a shade of purple, whilst the other was ashy pale; with no other symptom of excitement—anon, that cheek would become pale and the other flushed. Sometimes it would be only on the cheek; again, it would embrace the ear and run up to the median line of the nose its whole length, but not pass over it at any point, giving the countenance a very odd appearance. The skin is usually dry, rarely harsh. After five or six days the increase of temperature upon the surface generally, is scarcely to be observed. In many cases the temperature is natural or even less than natural. There is a very decided tendency in the knees and feet to become cool.

Sec. 2. *Rose Spots.*—I have never seen what I considered the "*rose colored lenticular spots,*" the typhoid eruption of Louis and Bartlett. I have seen petechiæ in the last stages usually a short time before death. Lest it should be supposed that this eruption has escaped my observation, because of negligence, I will state here, that I am fully confident of having used all due diligence. I have examined the body myself in cases of males, every day during the whole course of the disease. In female cases, I have had them examined in the same manner; and that in no few instances, until I became perfectly satisfied that it is an extremely rare occurrence in this vicinity. I have made the eruption, the special subject of conversation with many of my medical friends, and have never found one who had seen it, if the eruption upon the neck and face as described by Dr. Gano, before noticed, was not of that character. On the contrary, many of my friends have expressed their surprise that they had never observed it, though repeatedly sought for. Dr. Emison who saw this eruption in Philadelphia under Gerhard, and who is satisfied that when once seen it could not readily be mistaken or overlooked, has failed in discovering it. Dr. Prewett who had abundant opportunity to see this eruption in Boston, and who resides about twenty miles south of this, has seen but one case marked by this eruption. He, however, has seen but five or six cases since his return to Kentucky.

I have spoken of rose spots and petechiæ, and it may be well to define what I mean: By *the rose spot* is understood a small round or oval spòt, about a line in diameter, very slightly yet perceptibly raised above the adjacent skin, and of a bright red color; usually appearing successively during the second week, and continuing about a week; disappearing under pressure, and returning immediately; finally fading gradually without desquamation — generally not very numerous. Seated commonly on the breast and abdomen; occasionally upon the limbs. By *petechiæ* I mean dark red spots of different sizes, from half a line to two lines diameter, of irregular shape, though commonly round, not elevated; not disappearing on pressure, being in fact minute ecchymoses; usually numerous; situated on the body and extremities.

Since writing the above, I have been informed by Dr. Evans, who lives twelve miles north of Georgetown, that he has found the rose spots in about one half of the cases in his practice.*

SEC. 3. *Sudamina.*—I have found *sudamina* rather frequently; being present in perhaps a third of the cases. It is successive in its eruption, and goes off by leaving very thin scales. I have seen this eruption continued for several weeks after restoration to health, but most commonly it disappears about the time when convalescence is well established. I have seen one case at least, in which it did not appear until the patient was in *articulo mortis;* at least, I examined for it in the afternoon without finding

---

*July, 1850, I have seen within the last three weeks a crop of rose spots. It appeared upon my son, twenty-five years old, who had an attack of the fever in the early part of 1849, and another in the latter part of May last. He had recovered from this so far as to be able to ride for about a week, when, from indiscretion in eating, he had a relapse during which the rose spots appeared and continued four days, not more.

it; but at midnight I found the patient dying with a plentiful eruption upon the neck and lower part of his face.

SEC. 4. *Desquamation of the cuticle* has been observed something more frequently than sudamina. The scales of this desquamation are too large to have been occasioned by the debris of the sudamina. The first case which exhibited this symptom, was in a negro, and I was under the impression that it was the consequence of the rose colored lenticular spots, which the color of the skin had prevented my detecting; but in addition to the fact that I have not seen scales mentioned as a sequel of these spots, subsequent observations have satisfied me, that this symptom occurs frequently independent of any preceding eruption. Its most frequent seat is the chest and back, especially about the loins and sacrum.

---

### ARTICLE 7.

### RESPIRATORY SYSTEM.

Thoracic symptoms of a grave character I have rarely observed. I have frequently seen a short dry cough, such as is usually denominated stomach or worm cough. Occasionally there has been a slight rhonchus in respiration. There is frequently a somewhat hurried respiration. Towards the termination of fatal cases, the respiration becomes more irregular and sometimes hurried. The irregularity often is more marked in a sudden expiration than in anything else.

### ATRICLE 8.

### SEQUELÆ.

I have seen phthisis follow speedily upon typhoid fever in several instances. I have seen a very decidedly edematous condition of the face and limbs succeed the fever. This is most apt to be the case when a sufficient restraint upon the appetite has not been imposed. After severe attacks of this, as of other fevers, it is usual to have a considerable loss of hair. In three instances I have seen this amount to almost complete baldness, the patient losing every hair of his head, though not precisely at once. There is frequently considerable desquamation of the cuticle; but never to a degree so marked as occurs after scarlatina. I have seen one case in which a very painful affection of the tibia, accompanied by some tumefaction of the integuments, which also were exceedingly tender to the touch. A gentleman of my acquaintance had a very painful swelling of the leg: after the disappearance of the pain, the limb remained permanently enlarged.

I do not know that there is anything very important in these observations. I have not seen phthisis occur, except in persons strongly predisposed to it before the attack. We occasionally see an edematous condition of the body, or an inflammation of some particular part of the body (in popular language, a settling of the fever) follow bad cases of fever of any kind. I do not know that they are more apt to follow typhoid than other fevers. It seems to me that inflammation about the parotid gland has been less common than after bilious fever or dysentery, to say nothing of scarlatina.

